# Neuropathie optique compressive secondaire à une pseudo-tumeur inflammatoire

**DOI:** 10.11604/pamj.2015.21.226.7491

**Published:** 2015-07-30

**Authors:** Wafa Ammari, Olfa Berriche

**Affiliations:** 1 Service d'Ohtalmologie, Hôpital Taher Sfar, Mahdia, Tunisie; 2 Service de Médecine Interne, Hopital Taher Sfar, Mahdia, Tunisie

**Keywords:** Oeil, neuropathie, pseudotumeur, eye, neuropathy, pseudotumor

## Image en medicine

La neuropathie optique regroupe l'ensemble des lésions du nerf optique. Le diagnostic est habituellement clinique: diminution de l'acuité visuelle, altération de la vision des couleurs, déficit du champ visuel. Les étiologies sont multiples, l'origine compressive est rarement rapportée. La souffrance du nerf optique peut être dû dans ce cas soit à l'augmentation de la pression du liquide céphalo rachidien, soit à une compression par un processus intraorbitaire, intracanalaire ou intracrânien. Nous rapportons un cas rare de neuropathie optique d'origine compressive. Il s'agit d'un patient âgé de 43 ans ayant consulté pour une baisse de vision de l’œil droit. L'examen a retrouvé une acuité visuelle limitée à 50 cm, un œdème palpébral sans exophtalmie, un réflexe photomoteur afférent altéré et un œdème papillaire au fond d’œil. L'IRM orbitaire a mis en évidence des lésions en faveur de pseudotumeur inflammatoire engainant le nerf optique avec extension au niveau des fissures orbitaires. Le patient a été mis sous corticothérapie par voie générale avec bonne évolution.

**Figure 1:**
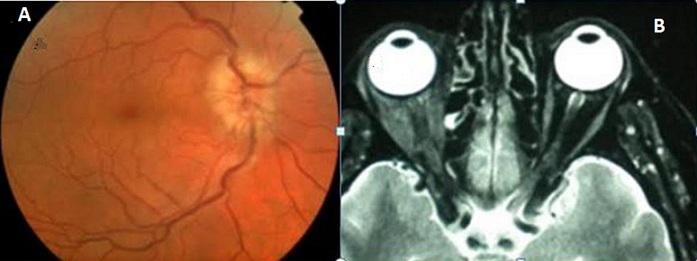
(A) photographie du FO droit montrant un oedème papillaire; (B) IRM orbitaire montrant un processus expansif intra orbitaire droit polylobé

